# Rapid Screening for Cognitive Impairment in Parkinson's Disease: A Pilot Study

**DOI:** 10.1155/2015/348063

**Published:** 2015-12-02

**Authors:** YanHong Dong, Way Inn Koay, Leonard Leong Litt Yeo, Christopher Li-Hsian Chen, Jing Xu, Raymond Chee Seong Seet, Erle Chuen Hian Lim

**Affiliations:** ^1^Department of Pharmacology, Yong Loo Lin School of Medicine, National University Health System, BLK MD3 Level 4 #04-01, 16 Medical Drive, Singapore 117600; ^2^Department of Medicine, Yong Loo Lin School of Medicine, National University Health System, 1E, Level 10, NUHS Tower Block, Kent Ridge Road, Singapore 119228; ^3^CHeBA, NPI, Euroa Centre, Prince of Wales Hospital, Barker Street, Randwick, NSW 2031, Australia; ^4^Division of Neurology, National University Hospital, 5 Lower Kent Ridge Road, Singapore 119074

## Abstract

*Aim*. This study sought to establish the discriminant validity of a rapid cognitive screen, that is, the National Institute of Neurological Disease and Stroke-Canadian Stroke Network (NINDS-CSN) 5-minute protocol, and compare its discriminant validity to the Montreal Cognitive Assessment (MoCA) and Mini Mental State Examination (MMSE) in detecting cognitive impairment (CI) in PD patients.* Methods*. One hundred and one PD patients were recruited from a movement disorders clinic in Singapore and they received the NINDS-CSN 5-minute protocol, MoCA, and MMSE. No cognitive impairment (NCI) was defined as Clinical Dementia Rating (CDR) = 0 and CI was defined as CDR ≥ 0.5.* Results*. Area under the receiver operating characteristic curve of NINDS-CSN 5-minute protocol was statistically equivalent to MoCA and larger than MMSE (0.86 versus 0.90, *P* = 0.07; 0.86 versus 0.76, *P* = 0.03). The sensitivity of NINDS-CSN 5-minute protocol (<9) was statistically equivalent to MoCA (<22) (0.77 versus 0.85, *P* = 0.13) and superior to MMSE (<24) (0.77 versus 0.52, *P* < 0.01) in detecting CI, while the specificity of NINDS-CSN 5-minute protocol (<9) was statistically equivalent to MoCA (<22) and MMSE (<24) (0.78 versus 0.88, *P* = 0.34).* Conclusion*. The NINDS-CSN 5-minute protocol is time expeditious while remaining statistically equivalent to MoCA and superior to MMSE and therefore is suitable for rapid cognitive screening of CI in PD patients.

## 1. Introduction

Parkinson's disease (PD) is the second most common chronic neurodegenerative disorder with a global prevalence of 0.5% to 4% in older adults aged ≥65 years [1]. The cumulative prevalence of PD dementia over 8 years is approximately 80% in patients whose mean PD duration was 9 years at baseline [2]. Approximately one-third of idiopathic PD patients in Singapore showed cognitive decline at an early stage of the disease [3]. Patients with PD are therefore a “population at risk” for cognitive impairment and require cognitive screening. Moreover, PD patients with cognitive impairment (CI) suffer from a reduced quality of life and increased caregiver burden and require long-term management [4]. Rapid screening for CI is an important step in achieving optimal long-term care for PD patients.

Several brief screening instruments are widely used in patients with PD, including the Mini Mental State Examination (MMSE) and Montreal Cognitive Assessment (MoCA). Although the MoCA was reported to be superior to the MMSE and well suited to screen for CI in PD patients [3], it takes approximately 10 minutes to administer in patients with subtle cognitive deficits and would take even longer time in patients with more severe cognitive impairment and those with bradyphrenia such as PD patients; therefore, it is less than ideal for rapid cognitive screening. The rapid bedside screening instrument should ideally take no more than 5 minutes and can be administered either in person or over the phone [5]. The National Institute of Neurological Disease and Stroke-Canadian Stroke Network (NINDS-CSN) recommended a 5-minute protocol drawn from the MoCA for rapid screening of poststroke vascular cognitive impairment (VCI) [5]. A number of studies reported that the NINDS-CSN 5-minute protocol was suitable for rapid screening in person or over the phone and equivalent to the MoCA in detecting vascular cognitive impairment and community-dwelling older adults with cerebrovascular risk factors [6–9]. In view of the shared cognitive pattern of “subcortical profile” such as bradyphrenia, frontal-executive and visuospatial deficits between poststroke VCI and CI in PD, the NINDS-CSN 5-minute protocol may be considered for rapid cognitive screening in PD patients. We therefore sought to assess the discriminant validity of the NINDS-CSN 5-minute protocol compared to the MoCA and MMSE in detecting CI in PD patients. Due to the previously established superiority of the MoCA to MMSE for cognitive screening in PD and the fact that the NINDS-CSN 5-minute protocol is drawn from the MoCA, we hypothesized that the NINDS-CSN 5-minute protocol is superior to the MMSE and equivalent to the MoCA for the detection of CI in PD patients.

## 2. Methods

### 2.1. Study Participants and Procedures

One hundred and one consecutive patients (≥55 years old) diagnosed with PD according to the London Brain Bank criteria [10] were consecutively seen and recruited from a movement disorders clinic at the National University Hospital, Singapore. Eligible patients had sufficient language skills in English, Chinese, or Malay for cognitive assessment. Patients with a major and active psychiatric illness, as well as those with any significant physical, visual, and/or hearing impairment that would impede the cognitive assessment, were excluded from the study. Whilst the patients were assessed in off state, they were taking different PD medications, including dopamine agonists (primarily ropinirole and bromocriptine), levodopa, anticholinergics such as benzhexol, neuroprotectants such as coenzyme q10 and selegiline, and COMT inhibitors such as entacapone. If they experienced behavioural or cognitive problems or hallucinations, they were only on levodopa. The severity of PD was measured by the Hoehn and Yahr scale [9], whilst motor function was determined by the Unified Parkinson's Disease Rating Scale (UPDRS) [10]. The MoCA and MMSE were administered by the trained research psychologist alternately or in a counterbalanced manner to avoid the influence of a standard repeated testing sequence on MMSE or MoCA scores. The MoCA was modified for Singaporean population such as replacing a test of letter fluency with semantic fluency (animals). Details of MoCA modification can be found in our previous paper [11]. The NINDS-CSN 5-minute protocol items comprising a 5-word recall (5 points), 6-item orientation (6 points), and verbal fluency (1 point if >10 words (animals) are generated in 60 seconds) were drawn from the MoCA [7]. The remainder of the MoCA test items include visuospatial/executive function (Trails B, Drawing a Cube, and a Clock), Naming (3 animals), Attention (digit span forward and backward, Vigilance, and Serial 7s), Language (Sentence Repetition), and Abstraction (Similarities). The score of the NINDS-CSN 5-minute protocol ranges from 0 to 12, with higher scores indicating a better cognitive performance. The gold standard criterion measure was the Clinical Dementia Rating (CDR), a widely used semistructured dementia staging instrument [12]. The trained research psychologist who completed CDR rating was blinded to the scores of the MoCA, NINDS-CSN 5-minute protocol, and MMSE. Cognitive impairment in PD patients was defined by the global CDR score and dichotomized into no cognitive impairment (NCI) (CDR = 0) and CI (CDR ≥ 0.5) groups. Patients with CI include those with questionable dementia (CDR = 0.5), definite mild dementia (CDR = 1), and moderate dementia (CDR = 2). This study was approved by the local ethics committee and conducted in conformity with the Declaration of Helsinki. Written informed consents were obtained from all patients and their informants.

### 2.2. Statistical Analyses

Data analysis was conducted using Statistical Package R version 3.2.0. Independent sample *t*-tests or Mann-Whitney *U* tests were used to compare between group differences of quantitative variables. Pearson's Chi-square test was conducted to compare between group differences of categorical variables. Receiver operating characteristic (ROC) curve analyses were conducted to establish the optimal cut-off points and discriminatory properties of the MoCA, MMSE, and NINDS-CSN 5-minute protocol in detecting CI in PD patients (CDR ≥ 0.5). Area under the curves (AUCs) of these test raw scores and scores adjusted by age and education were compared statistically [13]. The optimal cut-off points were chosen by Youden Index [14]. Additionally, McNemar's test was employed to compare sensitivity and specificity statistically.

## 3. Results

**Figure 1:**
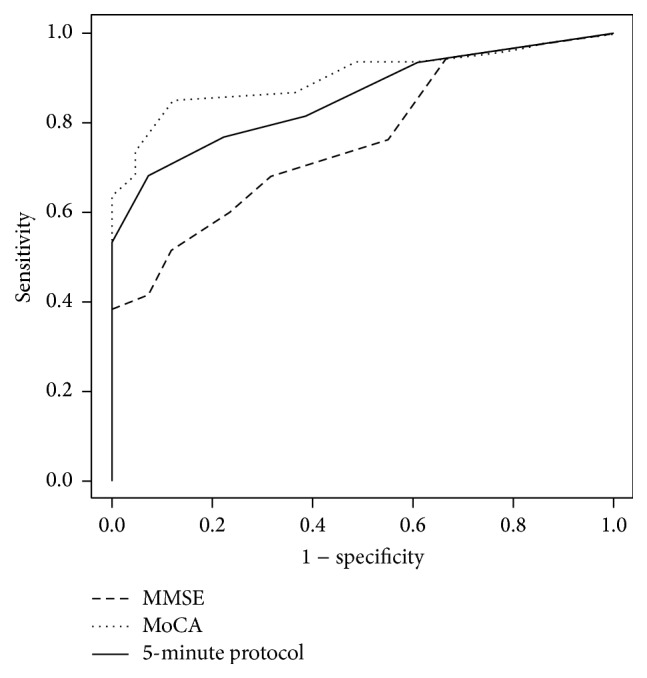
ROC curves for the MMSE, MoCA, and NINDS-CSN 5-minute protocol for detecting PD patients with CI.

Table 1 shows the characteristics of patients with NCI and CI. Of 101 PD patients recruited, a majority (60%, *n* = 60) had CI with the breakdown of CDR scores of 0.5 (*n* = 46), 1 (*n* = 9), and 2 (*n* = 5). Of these, a sizable number had dementia of mild-to-moderate severity; that is, CDR = 1 and 2 (23.3%, *n* = 14). PD patients with CI were older and had less education, longer duration of PD, and poorer performances in the MoCA, MMSE, and NINDS-CSN 5-minute protocol. They also had higher scores in the Unified Parkinson's Disease Rating Scale and Hoehn and Yahr scale but had lower scores in the Schwab and England Activities of Daily Living Scale than those with NCI.  Table 2 shows the discriminatory properties of the MoCA, MMSE, and NINDS-CSN 5-minute protocol. The ROC curves of the raw scores of MMSE, MoCA, and NINDS-CSN 5-minute protocol were plotted in Figure 1. The MoCA and NINDS-CSN 5-minute protocol had statistically larger AUCs than the MMSE for detecting CI from NCI (AUC (95% Confidence Interval): MoCA versus MMSE, 0.90 (0.84–0.96) versus 0.76 (0.67–0.85), *P* < 0.01, and 5-minute protocol versus MMSE, 0.86 (0.79–0.93) versus 0.76 (0.67–0.85), *P* = 0.03, resp.), while the AUCs of MoCA and NINDS-CSN 5-minute protocol were not statistically different (AUCs: MoCA versus 5-minute protocol, 0.90 versus 0.86, *P* = 0.07). ROC analyses repeated using age- and education-adjusted scores did not alter the results (data not shown). Moreover, at the optimal cut-off points, NINDS-CSN 5-minute protocol (<9) had acceptable sensitivity, which was statistically equivalent to MoCA (<22) but superior to MMSE (<24) (0.77 versus 0.85 and 0.52, *P* = 0.13 and *P* < 0.01, resp.), while the specificity of NINDS-CSN 5-minute protocol (<9) was statistically equivalent to MoCA (<22) and MMSE (<24) (0.78 versus 0.88 and 0.88 with *P* = 0.34 and *P* = 0.34, resp.).

## 4. Discussion

To the best of our knowledge, this is the first study to establish a rapid cognitive screen (NINDS-CSN 5-minute protocol) for the detection of CI in PD patients. The NINDS-CSN 5-minute protocol is statistically equivalent to the MoCA and both are superior to the MMSE in detecting CI. Our finding that the NINDS-CSN 5-minute protocol is statistically equivalent to the MoCA is consistent with a recent study of a population of patients with the stroke and transient ischemic attack (TIA), where cognitive impairment was defined by CDR ≥ 0.5. Similarly, the cut-off point of <9 for the NINDS-CSN 5-minute protocol established in the present study concurs with the cut-off point of <9 for the same test administered over the telephone in a previous stroke/TIA study (sensitivity: 0.77 versus 0.75; specificity: 0.78 versus 0.63) [7]. The NINDS-CSN 5-minute protocol has statistically equivalent discriminant indices (AUC, sensitivity, and specificity) to the MoCA and is therefore appropriate to detect CI in PD patients. The administration of MoCA generally takes approximately 10 minutes in patients with subtle cognitive deficits, which is not ideal for rapid cognitive screening in PD patients who may take an even longer time, due to bradykinesia, dysphonia, and bradyphrenia. The brevity and statistically equivalent discriminant ability of the NINDS-CSN 5-minute protocol appear to be well suited for rapid cognitive screening in PD.

Several study limitations should be acknowledged. First, the study sample of a total 101 patients is small and therefore may not provide adequate statistical power to detect AUC differences between MoCA and the NINDS-CSN 5-minute protocol. Moreover our patients had a mean PD duration of 5-6 years with a prevalence of 60% CI; therefore results in our study may not be generalizable to early PD patients who may have a lower prevalence of CI. Additionally, our PD patients were assessed in off state; therefore the results cannot be generalized to patients assessed in on state. Second, despite the fact that the difference is not statistically significant, based on higher sensitivity and specificity of the MoCA relative to NINDS-CSN 5-minute protocol, MoCA might be a better option for cognitive screening if there is no time constraint. However, under time pressure, clinicians might use the NINDS-CSN 5-minute protocol to corroborate their impression of patient's cognitive impairment and recommend these patients for additional formal neuropsychological evaluation to establish the diagnosis of CI in PD. Third, we did not record the time taken to administer the NINDS-CSN 5-minute protocol, as it was extracted from the MoCA. Although the administration time was not taken, the NINDS-CSN 5-minute protocol surely takes significantly shorter administration time compared to the MoCA because it only comprises 3 items of MoCA (5-word recall, 6-item orientation, and verbal fluency). Furthermore, the scores of NINDS-CSN 5-minute test were taken from the MoCA but not through independent administration of each test item. A more robust study methodology would administer the NINDS-CSN 5-minute protocol on its own rather than the item scores being extracted from the MoCA. It is possible that the difficulty level of individual test items in the NINDS-CSN 5-minute protocol may differ (i.e., be more or less difficult) if only the recall, orientation, and verbal fluency items were administered. Future study should include a larger sample of PD patients and examine the discriminant abilities of the NINDS-CSN 5-minute protocol administered independently from the MoCA for the detection of CI in PD. Finally, the NINDS-CSN 5-minute test protocol was developed based on expert opinion rather than empirical evidence [5]. The MoCA test items sensitive to cognitive pattern of “subcortical profile” (bradyphrenia and frontal-executive and visuospatial deficits) such as Trails B, Clock, and Serial 7s that may improve screening accuracy are not included in the NINDS-CSN 5-minute test. Therefore future empirical studies should develop a 5-minute test protocol drawn from the MoCA and validate it for rapid cognitive screening in PD.

## 5. Conclusion

In this study, we have examined a rapid cognitive screen, the NINDS-CSN 5-minute protocol drawn from the MoCA, and compared its discriminant validity to the MoCA and MMSE for the detection of CI in PD. The NINDS-CSN 5-minute protocol has shown acceptable sensitivity and specificity and appears to be statistically equivalent to the MoCA but more sensitive than MMSE in detecting CI in PD. Though both the MoCA and the NINDS-CSN 5-minute protocol are effective screens for CI in PD, the brevity of the NINDS-CSN 5-minute protocol is advantageous for it to be considered as a routine rapid cognitive screen for older PD patients who are at risk of cognitive impairment.

## Figures and Tables

**Table 1:** Population characteristics.

	CDR = 0		CDR ≥ 0.5		*P*
	*n* = 41		*n* = 60		
Age (mean, SD)	65.02	10.09	69.97	10.27	0.02
Years of education (mean, SD)	9.95	3.75	7.72	5.72	0.02
Female (number, %)	16.00	39%	26.00	43%	0.82
Ethnicity					0.07
Chinese (number, %)	35.00	85%	44.00	73%	
Malay (number, %)	2.00	5%	7.00	12%	
Indian (number, %)	2.00	5%	9.00	15%	
Others (number, %)	2.00	5%	0.00	0%	
MMSE (mean, SD)	26.22	2.13	21.93	5.16	<0.01
MoCA (mean, SD)	24.17	2.59	16.35	5.53	<0.01
NINDS-CSN 5-minute protocol (mean, SD)	9.83	1.48	6.50	2.64	<0.01
Duration of PD (mean, SD)	4.50	4.99	6.77	5.26	0.01
UPDRS (mean, SD)	20.80	10.99	27.97	15.37	0.01
Hoehn and Yahr scale					<0.01
1.0 (number, %)	20.00	49%	20.00	33%	
1.5 (number, %)	2.00	5%	1.00	2%	
2.0 (number, %)	18.00	44%	15.00	25%	
2.5 (number, %)	1.00	2%	8.00	13%	
3.0 (number, %)	0.00	0%	12.00	20%	
3.5 (number, %)	0.00	0%	3.00	5%	
4.0 (number, %)	0.00	0%	1.00	2%	
SEADL (mean, SD)	8.56	0.74	7.05	2.10	<0.01

Note: CDR = Clinical Dementia Rating; SD = Standard Deviation; MMSE = Mini Mental State Examination; MoCA = Montreal Cognitive Assessment; NINDS-CSN = National Institute of Neurological Disease and Stroke-Canadian Stroke Network; PD = Parkinson's disease; UPDRS = Unified Parkinson's Disease Rating Scale; SEADL = Schwab and England Activities of Daily Living Scale.

**Table 2:** Discriminant indices of MMSE, MoCA, and NINDS-CSN 5-minute protocol cut-off points in discriminating patients with CDR ≥ 0.5 from those with CDR = 0.

MMSE						MoCA						NINDS-CSN 5-minute protocol					
Cut-off	SEN	SPEC	PPV	NPV	Classified	Cut-off	SEN	SPEC	PPV	NPV	Classified	Cut-off	SEN	SPEC	PPV	NPV	Classified
25/26	0.68	0.68	0.76	0.60	0.68	23/24	0.92	0.54	0.74	0.81	0.76	10/11	0.93	0.39	0.69	0.80	0.71
24/25	0.58	0.78	0.80	0.56	0.66	22/23	0.87	0.63	0.78	0.76	0.77	9/10	0.82	0.61	0.75	0.69	0.73
**23/24** ^*∗*^	**0.52**	**0.88**	**0.86**	**0.55**	**0.66**	**21/22** ^*∗*^	**0.85**	**0.88**	**0.91**	**0.80**	**0.86**	**8/9** ^*∗*^	**0.77**	**0.78**	**0.84**	**0.70**	**0.77**
22/23	0.42	0.93	0.89	0.52	0.62	20/21	0.73	0.95	0.96	0.71	0.82	7/8	0.68	0.93	0.93	0.67	0.78
21/22	0.38	1.00	1.00	0.53	0.63	19/20	0.68	0.95	0.95	0.67	0.79	6/7	0.53	1.00	1.00	0.59	0.72

Note: MMSE = Mini Mental State Examination; MoCA = Montreal Cognitive Assessment; NINDS-CSN = National Institute of Neurological Disease and Stroke-Canadian Stroke Network; CDR = Clinical Dementia Rating; SEN = sensitivity; SPEC = specificity; PPV = positive predicted value; NPV = negative predicted value.

^*∗*^Optimal cut-off score.
